# Complete Genome Sequence of *Streptomyces* Phage Salutena

**DOI:** 10.1128/MRA.01308-20

**Published:** 2021-01-07

**Authors:** Jinha Kim, Tyler Higbee, James Clark, Tram Le, Mei Liu, Ben Burrowes

**Affiliations:** a Zachry Department of Civil and Environmental Engineering, Texas A&M University, College Station, Texas, USA; b Department of Biochemistry and Biophysics, Texas A&M University, College Station, Texas, USA; c Center for Phage Technology, Texas A&M University, College Station, Texas, USA; Queens College

## Abstract

*Streptomyces* are Gram-negative soil bacteria that can degrade lignin and synthesize antibiotics. Some species cause mycetoma, pneumonitis, and bloodstream infections. Here, we present the genome sequence of the *Streptomyces* sp. strain Mg1 phage Salutena, a siphovirus in the subfamily *Arquatrovirinae*. The genome is 51,993 bp, with 90 predicted protein-coding genes.

## ANNOUNCEMENT

*Streptomyces* spp. are Gram-positive, saprotrophic soil bacteria that can degrade lignin and synthesize industrial enzymes and unique antibiotics (1). *Streptomyces* sp. strain Mg1 secretes the antibiotic chalcomycin A, which can degrade Bacillus subtilis colonies (2). Furthermore, certain *Streptomyces* spp. can cause mycetoma and, in rare cases, pneumonitis and bloodstream infections (3). Here, we describe a novel phage, Salutena, that infects *Streptomyces* sp. Mg1.

Salutena was isolated from a South Jordan, UT, soil sample taken in August 2019 using the double-overlay agar technique (4). *Streptomyces* sp. Mg1 (provided by Paul Straight, Texas A&M University) was used as the host and grown on nutrient broth or agar at 30°C with 10 mM MgCl_2_, 8 mM Ca(NO_3_)_2_, and 0.5% glucose. Genomic DNA was purified as previously described (5) using a Wizard DNA cleanup kit (Promega). A paired-end sequence library was prepared with 300-bp inserts using the TruSeq Nano kit and was sequenced by an Illumina iSeq 100 instrument. A total of 367,310 reads were visualized (www.bioinformatics.babraham.ac.uk/projects/fastqc), manually trimmed with FastX Toolkit 0.0.14 (http://hannonlab.cshl.edu/fastx_toolkit/download.html), and assembled by SPAdes v3.5.0 with 86.8-fold contig coverage (6). Genome closure was confirmed by PCR and Sanger sequencing (forward primer, 5′-GATGTTCGTGGCGTTCAC-3′; reverse primer, 5′-ATCTTGAGCTGGCCGTAC-3′). Initial gene prediction was performed with GLIMMER v3 (7) and MetaGeneAnnotator v1.0 (8). The tRNA and rho-independent terminators were detected with ARAGORN v2.36 (9) and TransTermHP v2.09 (10), respectively. Gene functional predictions were made with InterProScan v5.33 by searching conserved functional domains (11). BLAST v2.9.0 (12) was used for similarity searches against the NCBI nonredundant, Swiss-Prot, and TrEMBL databases (13) (accessed 23 April 2020). TMHMM v2.0 predicted transmembrane domains at default settings (14). Whole-genome DNA sequence similarity was evaluated with progressiveMauve v2.4 (15). All annotation tools were hosted on the Galaxy platform by the Center for Phage Technology (https://cpt.tamu.edu/galaxy-pub) (16). HHpred v3.2.0 was used for validating functional annotation based on tertiary structure predictions of translated proteins (https://toolkit.tuebingen.mpg.de/tools/hhpred) (17). Default parameters were used for all software unless otherwise specified. After negative staining of the sample with 2% (wt/vol) uranyl acetate, phage morphology was evaluated by transmission electron microscopy (TEM) at the Texas A&M University Microscopy and Imaging Center, and the phage was determined to be a siphovirus (data not shown).

The Salutena genome is 51,993 bp, with a G+C content of 67.50% and a coding density of 92.68%. Based on the annotation results, 90 protein-coding genes were identified, of which 38 were assigned putative functions. Salutena shared highest nucleotide similarity (71.62%) with Streptomyces platensis MJ1A1 phage BartholomewSD (GenBank accession no. MK460245.1). Streptomyces azureus NRRL B-5410 phage Omar (MG593802.1) had the highest number of protein matches (77 proteins). Salutena is therefore a siphovirus of the subfamily *Arquatrovirinae*.

Only 1 tRNA and 1 rho-independent terminator were identified. Genes involved in phage morphogenesis, DNA packaging and replication, lysis, and transcription were also identified. A tape measure protein gene was found with tail assembly chaperone genes that are generated with a translational frameshift (18). One lysis protein gene, amidase endolysin, was identified. A GCN5-related *N*-acetyltransferase gene was identified by protein sequence (BLASTp) and predicted structural (HHpred) homology, indicating a potential regulatory posttranslational modification system through acetylation (19). No introns were identified. None of the assigned gene functions have been verified experimentally.

### Data availability.

The genome of Salutena has been deposited in GenBank under accession number MT708548.1. The associated BioProject, SRA, and BioSample accession numbers are PRJNA222858, SRR11558352, and SAMN14609629, respectively.

## References

[1] Harrison J, Studholme DJ. 2014. Recently published Streptomyces genome sequences. Microb Biotechnol 7:373–380. doi:10.1111/1751-7915.12143. 25100265 PMC4229319

[2] Barger SR, Hoefler BC, Cubillos-Ruiz A, Russell WK, Russell DH, Straight PD. 2012. Imaging secondary metabolism of Streptomyces sp. Mg1 during cellular lysis and colony degradation of competing Bacillus subtilis. Antonie Van Leeuwenhoek 102:435–445. doi:10.1007/s10482-012-9769-0. 22777252

[3] Kapadia M, Rolston KVI, Han XY. 2007. Invasive Streptomyces infections: six cases and literature review. Am J Clin Pathol 127:619–624. doi:10.1309/QJEBXP0BCGR54L15. 17369139

[4] Kropinski AM, Mazzocco A, Waddell TE, Lingohr E, Johnson RP. 2009. Enumeration of bacteriophages by double agar overlay plaque assay. Methods Mol Biol 501:69–76. doi:10.1007/978-1-60327-164-6_7. 19066811

[5] Summer EJ. 2009. Preparation of a phage DNA fragment library for whole genome shotgun sequencing. Methods Mol Biol 502:27–46. doi:10.1007/978-1-60327-565-1_4. 19082550

[6] Bankevich A, Nurk S, Antipov D, Gurevich AA, Dvorkin M, Kulikov AS, Lesin VM, Nikolenko SI, Pham S, Prjibelski AD, Pyshkin AV, Sirotkin AV, Vyahhi N, Tesler G, Alekseyev MA, Pevzner PA. 2012. SPAdes: a new genome assembly algorithm and its applications to single-cell sequencing. J Comput Biol 19:455–477. doi:10.1089/cmb.2012.0021. 22506599 PMC3342519

[7] Delcher AL, Harmon D, Kasif S, White O, Salzberg SL. 1999. Improved microbial gene identification with GLIMMER. Nucleic Acids Res 27:4636–4641. doi:10.1093/nar/27.23.4636. 10556321 PMC148753

[8] Noguchi H, Taniguchi T, Itoh T. 2008. MetaGeneAnnotator: detecting species-specific patterns of ribosomal binding site for precise gene prediction in anonymous prokaryotic and phage genomes. DNA Res 15:387–396. doi:10.1093/dnares/dsn027. 18940874 PMC2608843

[9] Laslett D, Canback B. 2004. ARAGORN, a program to detect tRNA genes and tmRNA genes in nucleotide sequences. Nucleic Acids Res 32:11–16. doi:10.1093/nar/gkh152. 14704338 PMC373265

[10] Kingsford CL, Ayanbule K, Salzberg SL. 2007. Rapid, accurate, computational discovery of Rho-independent transcription terminators illuminates their relationship to DNA uptake. Genome Biol 8:R22. doi:10.1186/gb-2007-8-2-r22. 17313685 PMC1852404

[11] Jones P, Binns D, Chang H-Y, Fraser M, Li W, McAnulla C, McWilliam H, Maslen J, Mitchell A, Nuka G, Pesseat S, Quinn AF, Sangrador-Vegas A, Scheremetjew M, Yong S-Y, Lopez R, Hunter S. 2014. InterProScan 5: genome-scale protein function classification. Bioinformatics 30:1236–1240. doi:10.1093/bioinformatics/btu031. 24451626 PMC3998142

[12] Camacho C, Coulouris G, Avagyan V, Ma N, Papadopoulos J, Bealer K, Madden TL. 2009. BLAST+: architecture and applications. BMC Bioinformatics 10:421. doi:10.1186/1471-2105-10-421. 20003500 PMC2803857

[13] The Uniprot Consortium. 2018. UniProt: the universal protein knowledgebase. Nucleic Acids Res 46:2699–2699. doi:10.1093/nar/gky092. 29425356 PMC5861450

[14] Krogh A, Larsson B, von Heijne G, Sonnhammer ELL. 2001. Predicting transmembrane protein topology with a hidden Markov model: application to complete genomes. J Mol Biol 305:567–580. doi:10.1006/jmbi.2000.4315. 11152613

[15] Darling AE, Mau B, Perna NT. 2010. progressiveMauve: multiple genome alignment with gene gain, loss and rearrangement. PLoS One 5:e11147. doi:10.1371/journal.pone.0011147. 20593022 PMC2892488

[16] Afgan E, Baker D, Batut B, van den Beek M, Bouvier D, Čech M, Chilton J, Clements D, Coraor N, Grüning BA, Guerler A, Hillman-Jackson J, Hiltemann S, Jalili V, Rasche H, Soranzo N, Goecks J, Taylor J, Nekrutenko A, Blankenberg D. 2018. The Galaxy platform for accessible, reproducible and collaborative biomedical analyses: 2018 update. Nucleic Acids Res 46:W537–W544. doi:10.1093/nar/gky379. 29790989 PMC6030816

[17] Zimmermann L, Stephens A, Nam S-Z, Rau D, Kübler J, Lozajic M, Gabler F, Söding J, Lupas AN, Alva V. 2018. A completely reimplemented MPI bioinformatics toolkit with a new HHpred server at its core. J Mol Biol 430:2237–2243. doi:10.1016/j.jmb.2017.12.007. 29258817

[18] Xu J, Hendrix RW, Duda RL. 2004. Conserved translational frameshift in dsDNA bacteriophage tail assembly genes. Mol Cell 16:11–21. doi:10.1016/j.molcel.2004.09.006. 15469818

[19] Favrot L, Blanchard JS, Vergnolle O. 2016. Bacterial GCN5-related N-acetyltransferases: from resistance to regulation. Biochemistry 55:989–1002. doi:10.1021/acs.biochem.5b01269. 26818562 PMC4795176

